# Socio-demographic determinants of mammography uptake among women of 40 years and above in Calabar, South-South, Nigeria: a cross-sectional study

**DOI:** 10.11604/pamj.2022.43.76.32683

**Published:** 2022-10-12

**Authors:** Glory Mbe Egom Nja, Grace Okaliwe, Grace Ofem Ibor, Isaac Olushola Ogunkola, Henshaw Uchechi Okoroiwu, Regina Idu Ejemot-Nwadiaro, Don Eliseo Lucero-Prisno III

**Affiliations:** 1 Department of Public Health, University of Calabar, Calabar, Nigeria,; 2 Department of Medical Laboratory Science, University of Calabar, Calabar, Nigeria,; 3 Department of Global Health and Development, London School of Hygiene and Tropical Medicine, London, United Kingdom

**Keywords:** Breast cancer, mammography uptake, socio-demographic determinants, women aged ≥40 years, Calabar, Nigeria

## Abstract

**Introduction:**

mammography has the potential for identifying high risk women with breast cancer. Early detection is important in reducing mortality and morbidity, and crucial for better prognosis. Mammography is poorly practiced in Nigeria. This study assessed the association between socio-demographic characteristics and uptake of mammography among women ≥40 years.

**Methods:**

a cross-sectional descriptive study design was employed and data was collected from 365 consenting participants in Calabar, Nigeria, using pre-tested questionnaire. SPSS Version 20 was used for data entry and analysis. The results were descriptively presented by frequencies and percentages. Pearson Chi-Square (χ^2^) analysis was performed to detect the association between variables at 5% level of significance (p-value of ≤0.05).

**Results:**

only 9.9% of participants had a mammography, majority 90.1% never had. Uptake was highest among respondents with tertiary education, married, Civil/Public Servants, and those in the high income level categorization. Educational status, marital status, occupation and age were not statistically significantly associated with mammography uptake (p>0.05). Only religious denominational affiliation (p = 0.02) and income level (p = 0.002) were statistically significantly associated with uptake. Barriers to uptake were poor knowledge about mammography (49.8%), psychosocial (37.8%), economic (17.1%) and health systems (11.5%). Key facilitators to uptake were encouragement/counselling by health workers (44.0%) and presence of breast problems (37.4%).

**Conclusion:**

mammography uptake in Calabar, Nigeria was extremely low. Therefore, regular awareness campaigns targeting women at faith-based settings, and provision of mammography screening services at subsidized rates will enhance knowledge level and uptake of mammography.

## Introduction

Breast cancer ranked second after lung cancer in all cancer cases and fourth in terms of mortality rates with over two million cases reported in 2018 globally. Breast cancer has been reported to be the most commonly diagnosed cancer in women, with about one in 4 of all new cancer cases diagnosed in women worldwide [1]. Breast cancer is fast becoming a serious public health challenge in low-resource countries. In sub-Saharan Africa, it is the commonest cancer in women [2]. It accounts for one in four cancer diagnosis and it is also responsible for one in five cancer deaths in women [3]. In 2020, an estimated 19.3 million new cases and almost 10.0 million cancer deaths were reported worldwide. Of this number, female breast cancer was reported to have surpassed lung cancer as the most commonly diagnosed cancer, with an estimated 2.3 million new cases worldwide [4]. Studies also show an increasing breast cancer incidence of 5% each year [5] and are projected to significantly increase further by the year 2030 [6]. Similarly, in Nigeria, breast cancer was reported to be the commonest cancer with 26,310 new cases (22%) and 11,564 (16.4%) mortality reported in 2018 [7].

Early detection of breast cancer is important in reducing mortality and morbidity [8]. Screening programmes allow for early diagnosis of cancer and are crucial for better prognosis and long-term survival [9]. Unfortunately, lack of public awareness on breast cancer screening and absence of organized screening programmes had made women to have late detection and presentations in Africa [10]. Early diagnosis and timely diagnostic follow up have been recognized as critical in reducing breast cancer morbidity, mortality and for improving overall outcomes [11].

Mammography is one of the breast screening services for early detection of breast cancer [6]. It is regarded as the gold standard for breast cancer screening in developed countries [12], but yet to attain such status in developing countries. It has been recognized to reduce the risk of dying from breast cancer by >30% [8]. It is recommended that women should initiate yearly mammograms at the age of 40 years [1]. Despite the high sensitivity and specificity of mammography in detecting early breast cancer and its benefits, the level of utilization/uptake remains low in low and middle income countries [6,12-16]. Some of the reasons advanced by these authors were the lack of knowledge on mammography, cost of services, high technology equipment, and the dearth of experts required for the screening. Even with the availability of mammography screening tools, women's participation in the screening services in Nigeria is poor [6]. There is paucity of information on the role of socioeconomic factors in contributing to the low uptake of mammography, yet, socioeconomic factors have been touted to have strong influence on health services utilization and overall health outcomes [11,17,18]. This study therefore assessed the association between socio-demographic/socio-economic characteristics and uptake of mammography as a breast screening service among women aged 40 years and above in Calabar, South-South Nigeria.

## Methods

**Study setting and population:** the study setting was Calabar Municipality, one of the 18 Local Government Areas of Cross River State; a State in the South-South region of Nigeria. Most of the State lies within the tropical rain forest belt of Nigeria. It lies between latitude 40 28' and 60 55' north of the equator and longitude 70 50' and 90 28' east of the Greenwich meridian. Calabar Municipality consists of 10 political wards (the smallest administrative unit). Its multi-cultural nature attracts people from all over Nigeria but the indigenous tribes are the *Quas* and the *Efiks*. Located within Calabar Municipality are several healthcare facilities including one tertiary health facility, two secondary health facilities (Navy Reference Hospital and General Hospital), 29 primary healthcare centres and about 18 privately owned health facilities. There were three functional Mammography screening centres in Calabar Municipality at the time of this study, domiciled in Asi Ukpo Diagnostics and Medical Centre, Navy Reference Hospital, and Arubah Specialist Hospital and Diagnostics. The study population comprised women aged 40 years and above residing in Calabar Municipality. Women who were aged below 40 years were excluded from the study.

**Study design and Sample size determination:** a descriptive cross-sectional study design using quantitative method of data collection was used in this study. The sample size of 411 women aged 40 years and above was determined using Bluman formula for dichotomous descriptive study, [19] employing the 40.5% awareness rate for mammography [20] at 95% confidence interval and 5% precision, while assuming a non-response rate of 10%.

**Sampling method:** purposive sampling technique was adopted in selecting the study setting. The selection was based on the availability of Mammography screening facilities in some health facilities in the geographical area of the study. A multi-stage sampling technique was thereafter used to randomly select 411 women aged 40 years and above for the study. The first stage involved the simple random selection of five out of the 10 political wards in Calabar Municipality by drawing a ballot without replacement. The second stage consisted of selection of streets from the selected political wards. A sampling frame containing all the streets in each of the selected wards was prepared. The names of the streets were written out on pieces of paper, folded and deposited in five separate containers. A research assistant was asked to pick six pieces of paper from each of the containers without replacement. Thirty streets were subsequently randomly selected through this process from the five selected political wards. The systematic sampling method used at third stage employed a fixed interval and a random starting point to select households from the selected streets, where the interval (K) was determined by dividing the total number of households by the desired number of households to be sampled. A total of 14 households from each of the 30 selected streets were chosen, giving a total of 420 households. Simple random sampling was adopted in the fourth stage in the selection of at least one eligible respondent from each of the randomly selected households. Through this method, a total of 411 women aged 40 years and above were recruited for the study.

**Method of data analysis:** out of the 411 questionnaire copies distributed to respondents, only 365 were retrieved giving a response rate of 88.8%. Cleaning, coding and analysis of data obtained from this study were done using Statistical Package for Social Sciences (SPSS version 20). Data was presented as frequencies, percentages, tables and charts. Chi-square (χ^2^) or Fisher's exact test was used to determine existence of associations between variables at 5% level of significance. P-value of <0.05 was considered as statistically significant.

**Ethical considerations:** ethical approval for conduct of this study was sought and obtained from the Ethics Committee of the Department of Public Health, University of Calabar, Nigeria, following a thorough review of the research proposal. A copy of the approval was tendered to the Leaders of the Political Wards where the study was conducted. The study participants were informed of the purpose of the research, and verbal informed consent obtained from the respondents before administering the questionnaire. All the respondents were assured of anonymity and confidentiality of information volunteered. They were also informed that participation in this study was voluntary and that participants were at liberty to discontinue from the study if they no longer felt comfortable with the issues raised in the questionnaire.

**Consent to participate:** informed consent was obtained from all the participants. The respondents were assured of anonymity and confidentiality of the information they provided.

## Results

**Socio-demographic description of respondents:** the socio-demographic characteristics of the respondents are shown in Table 1. One third of the total study participants 121 (33.1%) were aged 40-44 years, followed by 108 (29.6%) aged 45-49 years, and 19 (5.2%) respondents who were 60 years and above. A greater proportion 263 (72.0%) were married, while 102 (28.0%) were single: singleness comprised those who were never married 40 (11.0%), widowed 48 (13.1%), separated 9 (2.4%) and divorced 5 (1.4%). The educational background of the respondents showed that the majority 231 (63.2%) were graduates with tertiary education, while 14 (3.5%) had no formal education. Almost all the respondents 363 (99.4%) were Christians, with most 173 (47.3%) who were Protestants. More than half 214 (58.6%) of the total study participants were civil/public servants, 86 (23.6%) were business women, and 23 (6.3%) health workers. A small proportion 27 (7.4%) had no regular source of income, including those who were unemployed 11 (3.0%) and those who had retired from service 16 (4.4%).

**Table 1:** description of respondents by socio-demographic characteristics

Characteristics	Frequency ( n = 365)	Percentage
**Age (in Years)**		
40 - 44	121	33.1
45 - 49	108	29.6
50 - 54	82	22.5
55 - 59	35	9.6
> 60	19	5.2
**Marital status**		
Married	263	72.0
Single	40	11.0
Widowed	48	13.1
Divorced	5	1.4
Separated	9	2.4
**Occupation**		
Civil/Public servant	214	58.6
Business	86	23.6
Farming	15	4.1
Unemployed	11	3.0
Retired	16	4.3
Health worker	23	6.3
**Educational status**		
No formal education	14	3.8
Completed primary school	34	9.3
Completed secondary school	86	23.5
Tertiary Education	231	63.2
#Monthly income level (Naira)*		
Low income (< 30000)	133	36.4
Mid income (31000-70000)	98	26.4
High income (≥71000 )	134	36.7
**Religion**		
Christianity	363	99.4
Islam	2	0.6
**Denominational affiliation**		
Pentecostals	173	47.3
Catholics	59	16.1
Deeper Life Bible Church	65	17.9
Jehovah's Witnesses	3	0.8
Protestants	65	17.9

* Naira (Nigerian currency), # Income level categorizations are based on the Nigerian national minimum wage of ₦30000 per month, at the current exchange rate of one USD to ₦474.00

**Socio-economic description of the respondents:** all the study participants were categorized into three income levels (low, mid, and high). The income level categorizations are based on the Nigerian national minimum wage of ₦30,000 (63 USD) per month, at the exchange rate of one United States Dollar to ₦474.00. Less than one third of the total respondents 134 (36.7%) and 133 (36.4%) were in the high income level category (≥ ₦71,000) and low income level category (≥ ₦30,000) respectively. Only 98 (26.9%) were in the mid income level category (₦31,000 - ₦70,000) (Table 1).

**Uptake of mammography screening among respondents:** only 36 (9.9%) of respondents had a mammography screening, while the greater proportion 329 (90.1%) had never done a mammography screening. Results further showed that of the 36 respondents who had a mammography screening, more than half 22 (61%) had it once, while 4 (11%) had it more than three times.

**Association between respondents' socio-demographic/socio-economic characteristics and uptake of mammography:** descriptive analysis of data showed that uptake of mammography screening was highest among respondents with Tertiary level of education 30 (83.3%); those who were married 26 (72.2%); Civil/Public Servants 25 (69.4%); and those in the 40-44 years' age bracket 15 (41.7%). Furthermore, results show that Christians who were of the Pentecostals denomination 17 (47.2%) were more likely to have a mammography screening than others. Uptake of mammography screening was lowest among respondents aged 55 years and above 4 (11.1%); the singles 10 (27.8%), peasant farmers and those with no formal education 1(2.8%). Similarly, mammography uptake was lowest among respondents in the low income level category 5 (13.9%) compared to those in the mid income level 8 (22.2%) and high income level 23 (63.9%) respectively. A total of 23 (6.3%) health workers participated in this study, out of which only 4 (17.4%) had a mammography screening, while 19 (82.6%) did not. Chi-square analysis of respondents' socio-demographic characteristics (educational status, age, marital status, and occupation) and uptake of mammography were not statistically significantly associated (P>0.05), while religious denominational affiliation (P = 0.02) and monthly income (P = 0.002) were statistically significantly associated with mammography uptake (Table 2).

**Table 2:** link between respondents' socio-demographic characteristics and mammography uptake

Characteristics	Uptake of Mammography			Test Statistics	P-value
	Have Had (n = 36)	Have Not (n = 329)	Total (N = 365)		
**Age (in years)**					
40-44	15 (41.7)	106 (32.2)	121 (33.1)	χ^2^ Cal. = 5.62 df = 4	p = 0.22 (p >0.05)
45-49	7 (19.4)	101 (30.7)	108 (29.6)		
50-54	6 (16.7)	76 (23.1)	82 (22.5)		
55-59	4 (11.1)	31 (9.4)	35 (9.6)		
>60	4 (11.1)	15 (4.6)	19 (5.2)		
**Marital status**					
*Singleness	10 (27.8)	92 (28.0)	102 (27.9)	χ^2^ Cal. = 0.0006 df = 1	P = 0.98 (p>0.05)
Married	26 (72.2)	237 (72.0)	263 (72.1)		
**Occupation**					
Civil/Public Servant	25 (69.4)	189 (57.4)	214 (58.6)	χ^2^ Cal. = 6.413 df = 4	P= 0.17 (P>0.05)
Business	3 (8.3)	83 (25.2)	86 (23.6)		
Farming	1 (2.8)	14 (4.3)	15 (4.1)		
#Unemployed	3 (8.3)	24 (7.2)	27 (7.4)		
Health worker	4 (11.1)	19 (5.8)	23 (6.3)		
**Educational status**					
No formal education	1 (2.8)	13 (4.0)	14 (3.8)	χ^2^ Cal. = 7.11 df = 3	p=0.07 (p>0.05)
Completed primary School	1 (2.8)	33 (10.0)	34 (9.3)		
Completed secondary School	4 (11.1)	82 (25.0)	86 (23.5)		
Tertiary education	30 (83.3)	201 (60.0)	231 (63.2)		
**Monthly income level (Naira)ꚛ**					
Low income	5 (13.9)	126 (38.3)	131 (35.9)	χ^2^ Cal. = 18.47 df = 2	p = 0.002 (p<0.05)
Mid income	8 (22.2)	92 (28.0)	100 (27.4)		
High income	23 (63.9)	111 (33.7)	134 (36.7)		
Religion					
Christianity	35 (97.2)	328 (99.7)	363 (99.4)	χ^2^ Cal. = 3.64 df = 1	P = 0.06 (P>0.05)
Islam	1 (2.8)	1(0.3)	2 (0.6)		
**Denominational affiliation**					
Pentecostals	17 (47.2)	156 (47.4)	173 (47.4)	χ^2^ Cal. =11.27 df = 4	P= 0.02 (P<0.05)
Catholics	6 (16.7)	53 (16.1)	59 (16.2)		
Deeper Life Bible Church	5 (13.9)	60 (18.2)	65 (17.8)		
Jehovah's Witnesses	2 (5.6)	1 (0.3)	3 (0.8)		
Protestants	6(16.7)	59 (18.0)	65 (17.8)		

* Frequency of respondents who were single (never married, divorced, widowed, separated), ꚛ₦(Nigerian currency - Naira) # Frequency of respondents with no regular source of income (not employed, retired), Figures in parenthesis are percentages, df (Degree of Freedom)

**Barriers to uptake of mammography screening:** barriers to uptake of mammography screening among respondents were grouped under four main categories: knowledge, psychosocial, economic and health systems barriers. Among the total study participants who have never had a mammography screening 329 (90.1%), poor knowledge about mammography as a breast cancer screening service 164 (49.8%) accounted for the major barrier to uptake. This was closely followed by psychosocial barriers 125 (37.8%) with myths about breast cancer (perceived non-susceptibility to developing breast cancer) 70 (21.3%), self-procrastination (delay) in uptake of mammography screening 24 (7.2%), and embarrassment from the procedure 16 (4.8%) as contributing factors. Economic barriers 54 (17.15) included the lack of transportation to mammography screening centres 33 (10.2%) and perceived cost of mammography screening services 21 (6.9%). Absence of mammography screening facilities 22 (6.7%) and distance from where respondents lived to mammography screening facilities 16 (4.8%) were the contributing factors to the Health systems barriers to mammography uptake 38 (11.5%). Furthermore, 62 (18.8%) respondents said they would never have a mammography screening for any reason (Table 3).

**Table 3:** barrier to and facilitators of uptake of mammography among respondents

Factors	Statements	#Frequency* (n = 329)	Percentage
**Barriers categorization**			
Knowledge barrier (n = 164; 49.8%)	I don't know what mammography is all about	164	49.8
Health system barrier (n = 38; 11.5%)	No health facility for mammography screening	22	6.7
	The screening centre is far from where I live	16	4,8
Economic barrier (n = 54; 17.1%)	Mammography screening is expensive	21	6.9
	I don't have money for transportation to go and do the test	33	10.2
Psychosocial barriers (n = 125; 37.8%)	It is painful and frightening	8	2.4
	Fear of being diagnosed with breast cancer	5	1.5
	The procedure is embarrassing	16	4.8
	I will never have breast cancer (myths)	70	21.3
	Spousal disapproval	2	0.6
	Self-procrastination	24	7.2
Others (n = 62; 18.8%)	I will never go for mammography for whatever reason	62	18.8
**Facilitators categorization**			
Support from significant others (n = 152; 46.1%)	Encouragement and counselling from a doctor or other healthcare providers	145	44.4
	Advice from friends	7	2.1
Perceived susceptibility to breast cancer (n = 142; 43.6%)	Presence of breast problem	123	37.4
	History of breast cancer among family and friends	19	5.8
Awareness/knowledge (n = 18; 5.4%)	Health/awareness campaign about breast cancer	18	5.4
Economic factor (n = 47; 14.2%)	Availability and affordability of mammography	31	9.4
	Others	16	4.8

# Frequency of respondents who have not had mammography screening *Multiple responses

**Facilitators/enablers of uptake of mammography screening among respondents:** respondents who did not have a mammography screening 329 (90.1%) enumerated several probable facilitators/enablers of mammography uptake (Table 3) Most 145 (44.0%) said they would have a mammography screening if they received encouragement and counselling from the healthcare workers, while 123 (37.4%) said they would have a mammography screening if they had any form of breast problem. Other enablers of mammography uptake according to the respondents were availability/affordability of mammography screening services 31 (9.4%); history of breast cancer among family members and friends 19 (5.8%); and health campaigns on mammography screening 18 (5.4%).

## Discussion

Understanding factors that guide the development of comprehensive breast cancer control plan is highly imperative to reducing its rising public health burden particularly in resource poor countries. This study thus sought to determine the associations between socio-demographic factors and the uptake of mammography, the main stay of early breast cancer detection amongst women 40 years and above. The findings of this study show that uptake of mammography was quite low (9.9%). This is similar to the report of the findings by Akwo *et al*. [15] who also reported a low mammography uptake of 15.4% in 2019 in Cross River State, Nigeria. However, in comparison with the study by Akwo *et al*. [15], the findings in this present study in the same study setting, showed a marked decline in a short space of time in terms of mammography uptake among women in Cross River State, Nigeria. Low mammography uptake has similarly been reported in other parts of Nigeria. For instance, a study conducted in 2011 in Osogbo, South Western Nigeria by Bello *et al*. [21] reported 22.9% uptake among female nurses and 15.0% among non-health professionals. In 2019, Olasehinde *et al*. [12] reported an even lower level of mammography uptake of 2.8% in Ife Central and 1.8% in Iwo in the same geopolitical zone of Nigeria. In South Eastern Nigeria, Madubogwu *et al*. [22] reported very low mammography uptake of 1.9% in Nnewi, Nigeria. Similarly, low mammography uptake has been reported in the Northern part of Nigeria as exemplified by the findings of Gali [23] among female healthcare workers and female non-health workers in the University of Maiduguri, Borno State, Nigeria. The aforementioned reports also show the prevailing low uptake of mammography in other parts of Nigeria [3,6].

These abysmal statistics were equally reported in other African countries (Uganda, Egypt) [13,23-25] as well as in low-and middle-income countries (LMICs) (Malaysia, Brazil, Tehran, Iran) [14,26-28]. However, these findings contrast greatly with the rate of mammography uptake in developed countries [8,29] where it is regarded as the gold standard in breast cancer diagnosis [1,11,12]. This brings to the fore the breast cancer control differential between the developing and developed countries, rooted in lack of recognized national screening programs, coupled with overall weak health systems in the former [11,12,16,21,25]. These have been touted as contributory factors to the rising breast cancer burden in LMICs with its concomitant effects on breast cancer outcomes [3,6].

While the lack of established national screening programme may be suggested as a major factor limiting mammography uptake, many authors have implicated poor knowledge about breast cancer and mammography, particularly in developing countries [12,15,16,25]. Good knowledge has been recognized to positively influence not only the adoption of health-seeking behaviour, but also reduces myths and misconceptions about disease conditions, thereby facilitating a reduction in disease burden [6,20,25]. The overall low uptake of mammography in this study ties closely to the high proportion of respondents that reported lack of knowledge about mammography as a major barrier to uptake. This thus presents justification why awareness about breast cancer should be at the core of its control programmes [12,16,18,20,21].

The findings of this study show that marital status was not statistically significantly associated with mammography uptake, in spite of the fact that the greater proportion of the respondents was married. Ejemot-Nwadiaro *et al*. [18] and Ikeda *et al*. [30] also observed that marital status was not independently associated with disease morbidity and mortality. It is generally believed that those who are married tend to utilize health services better than the singles, probably due to the support and reinforcement of addressing health needs by significant others [31]. This notion is supported by the findings in this study where significant proportion of respondents identified encouragement by family members as a potential facilitator of mammography uptake. These findings also agree with the study by Akwo *et al*. [15] who observed that many women would consider having a mammography if instructed by their husbands.

Whereas Ahmadian *et al*. [28], Fontana & Bischoff [29] and Ifeanyichukwu [32] noted a significant association between educational status and uptake of mammography in their studies, our study did not, even though almost two-third of this study respondents had attained tertiary level of education. The implication of this finding is that deliberate awareness and sensitization programmes on breast cancer and mammography need to be mounted to optimize the benefits of education in influencing uptake of healthcare services [11,17], since higher level of education did not translate to uptake of mammography. Another dimension for justifying the need for targeted education as core element in effective breast cancer control programmes, as supported by findings of this study, is that it would debunk ignorance and myths associated with breast cancer, since significant proportion of this study respondents identified psychosocial factors as barrier to mammography uptake. This notion corroborates the findings in other studies where low uptake of mammography was attributed to embarrassment from the procedure, fear from painful sensation, fear of cancer diagnosis, fear from exposure to radiation and spousal refusal [23,31]. Furthermore, the need for awareness creation in breast cancer control programmes is underscored by a significant proportion of respondents in this study who reported that they would uptake a mammography screening if encouraged by healthcare providers. This finding is in consonance with the results of Walder *et al*. [10], Okaliwe *et al*. [16] and Ba *et al*. [25] that reported healthcare workers' role in breast cancer control. Also, the findings in this study identified perceived susceptibility to breast cancer as one of the facilitators for mammography uptake. Healthcare workers therefore have a critical role to play in the uptake of mammography, as they stand as first-line referrals for breast cancer screening.

In this present study, religious denominational affiliation was observed to be statistically significantly associated with uptake of mammography screening, though the frequencies of our religious denominational affiliation variable were relatively small to make strong statement. Nonetheless, faith and religion have been shown to have strong correlation with uptake of health services [31,33]. Chatters [33] reported that the overall better physical health status was associated with higher levels of religious involvement. Religion or faith tend to exert influence in building hope for survival and trust in health treatment, as such it strengthens health promotion activities [31]. The foregoing thus suggests that taking health promotion activities to places of religious worship could potentially improve uptake of health services including mammography. The flip side of influence of religion may reflect in over dependence on the belief that faith/religion could prevent the occurrence of undesirable health conditions which may reinforce myths that have potentials for limiting mammography uptake [31], which was supported by our observations in this study. Thus, how religious denomination affiliation influences uptake of mammography warrants further investigations.

Low income was statistically significantly associated with low mammography uptake in this study, which is not entirely surprising as income and financial factors have been implicated as huge barriers to utilization of healthcare services [6,14,15,17,18,25,29,31]. In addition, substantial number of Nigerians live below the poverty line with little disposable income to get by, playing out in a cumulative manner to financial incapability and inequities in access to healthcare [3,6,11,16]. This low income association with mammography uptake is in agreement with significant proportion of respondents in this study reporting cost of mammography services and transportation to screening centres as barriers to mammography uptake. Low income and financial factors exert influence on overall health outcomes, in that they may limit not only access, but could prompt negative health-seeking behaviours, place undue pressure on already weak health systems and can cause overall delay in diagnosis and treatment which carry in itself a huge life-long psychosocial and financial burden [11,17,18,21,25].

## Conclusion

Mammography uptake in Calabar, South-South Nigeria was abysmally low. A considerable increase in mammography uptake could be achieved through increase in education and awareness programmes on benefits of screening and overall knowledge on breast cancer, buoying support from families and loved ones, improvement in access, availability and affordability of mammography services at subsidized rates, and targeting faith-based settings for breast cancer awareness creation, would optimize the benefits of faith/religion in influencing health outcomes. Understanding how these factors interact singly or in combination, represents critical steps not only for effective breast cancer care programme planning but for improving its overall outcomes.

### What is known about this topic

- Breast cancer remains the most frequently diagnosed cancers in women and the leading cause of cancer mortality worldwide; mammography is one of the breast screening services for early detection of breast cancer;
- In sub-Saharan Africa, it is the most common cancer in women; early breast cancer testing/screening has been recognized to improve prognosis and save lives;
- There is huge breast cancer control differential between the developing and developed countries, rooted in lack of recognized national screening programs, coupled with overall weak health systems in the former.

### What this study adds

- Low uptake of mammography services still persist in this setting; poor knowledge about breast cancer and mammography limits mammography uptake;
- This study did not observe significant association between educational status and mammography uptake;
- The implication of the above is that deliberate awareness and sensitization programmes on breast cancer and mammography are imperatives.

## References

[1] (2018). International Agency for research on cancer: (e.d).

[2] Akarolo-Anthony SN, Ogundiran TO, Adebamowo CA (2010). Emerging breast cancer epidemic: evidence from Africa. Breast Cancer Research.

[3] Azubuike SO, Muirhead C, Hayes L, McNally R (2018). Rising global burden of breast cancer: the case of sub-Saharan Africa (with emphasis on Nigeria) and implications for regional development: A review. World J Surg Onc.

[4] Sung H, Ferlay J, Siegle RL, Laversanne M, Soerjomataram I, Jemal A (2021). Global Cancer Statistics 2020: GLOBOCAN Estimates of incidence and mortality for 36 cancers in 185 countries. CA Cancer J Clin.

[5] Adebamowo CA, Ajayi OO (2000). Breast cancer in Nigeria. West Afr J Med.

[6] Lawal O, Murphy F, Hogg P, Irurhe N, Nightingale J (2015). Mammography screening in Nigeria-A critical comparison to other countries. Radiography.

[7] The Global Cancer Observatory (GLOBOCAN) Nigeria: Globocan 2019. International Agency for Research on Cancer & World Health Organization (WHO).

[8] Kearney AJ, Murray M (2009). Breast cancer screening recommendations: is mammography the only answer?. J Midwifery Womens Health.

[9] Gucuk S, Uyeturk U (2013). Effect of direct education on breast self examination awareness and practice among women in Bolu, Turkey. Asian Pac J Cancer Prev.

[10] Wadler BM, Judge CM, Prout M, Allen JD, Geller AC (2011). Improving breast control via the use of community health workers in South Africa: A Critical Review. J Oncol.

[11] Akinyemiju T, Ogunsina K, Sakhuja S, Ogbhodo V, Braithwaite D (2016). Life-course socioeconomic status and breast and cervical cancer screening: analysis of the WHO´s Study on Global Ageing and Adult Health (SAGE). BMJ Open.

[12] Olasehinde O, Alatise OI, Arowolo OA, Mango VL, Olajide OS, Omisore AD (2019). Barriers to mammography screening in Nigeria: A survey of two communities with different access to screening facilities. Eur J Cancer Care (Engl).

[13] Elsie KM, Gonzaga MA, Francis B, Michael KG, Rebecca N, Rosemary BK (2010). Current knowledge, attitudes and practices of women on breast cancer and mammography at Mulago hospital. Pan Afr Med J.

[14] Al-Naggar RA, Bobryshev YV (2012). Practice and Barriers of mammography among Malaysian women in the general population. Asian Pac J Cancer Prev.

[15] Akwo JD, Erim AE, Ikamaise VC, Archibong B, Ekpo EU (2019). Transforming screening uptake in Low-resource and under-informed populations: A preliminary study of factors affecting women´s decisions to uptake screening. J Med Imaging Radiat Sci.

[16] Okaliwe G, Nja GME, Ogunkola IO, Ejemot-Nwadiaro RI, Lucero-Prisno III DE Breast cancer knowledge and mammography uptake among women aged 40 years and above in Calabar Municipality, Nigeria. Asian Journal of Medicine and Health.

[17] Downing A, Prakash K, Gilthorpe MS, Mikeljevic JS, Forman D (2007). Socioeconomic background in relation to stage at diagnosis, treatment and survival in women with breast cancer. Br J Cancer.

[18] Ejemot-Nwadiaro RI, Nja GM, Itam EH, Ezedinachi EN (2020). Socio-demographic and nutritional status correlates in pulmonary tuberculosis patients in Calabar, Nigeria. Asian Journal of Medicine and Health.

[19] Bluman AG (2004). An introduction to medical statistics.

[20] Amoran OE, Toyobo TO, Fatugase OK (2014). Breast cancer screening awareness and practice among women in Sagamu Local Government Area, South-Western Nigeria: A community-based study. British Journal of Applied Science and Technology.

[21] Bello TO, Olugbenga-Bello AI, Ogunsola AS, Adeoti ML, Ojemakinde OM (2011). knowledge and practice of breast cancer screening among female nurses and lay women in Osogbo Nigeria. West Afr J Med.

[22] Madubogwu CI, Egwuonwu AO, Madubogwu NU, Njelita IA (2017). Breast cancer screening practices amongst female health workers in Nnewi, Nigeria. J Cancer Res Ther.

[23] Gali BM (2013). Breast cancer awareness and screening practices among females health worker of University of Maiduguri Teaching Hospital. Borno Medical Journal.

[24] El-Nasr EMS (2017). Breast cancer risk factors and screening practices among women attending family health centres in Cairo Governorate. Journal of Nursing and Health Science.

[25] Ba DM, Ssentongo P, Agbese E, Yang Y, Cisse R, Diakite B (2020). Prevalence and determinants of breast cancer screening in four sub-Saharan African countries: a population based study. BMJ Open.

[26] Marinho LAB, Cecatti JG, Osis MJD, Gurgel MSC (2008). Knowledge, Attitude and Practice of Mammography among women users of public health services in Brazil. Rev Saude Publica.

[27] Bener A, R Alwash, Miller CJ, Denic S, Dunn EV (2001). Knowledge, attitudes, and practices related to breast cancer screening: A survey of Arabic women. J Cancer Educ.

[28] Ahmadian M, Samah AA, Redzuan M, Emby Z (2012). Predictors of mammography screening among Iranian women attending out-6patient clinics in Tehran, Iran. Asian Pac J Cancer Prev.

[29] Fontana M, Bischoff A (2008). Uptake of breast cancer screening measures among immigrant and Swiss women in Switzerland. Swiss Med Wkly.

[30] Ikeda A, Iso H, Toyoshima H, Fujino Y, Mizoue T, Yoshimura T (2007). Marital status and mortality among Japanese men and women: The Japan collaborative cohort study. BMC Public Health.

[31] Lambert M, Mendenhall E, Kim AW, Cubasch H, Joffe M, Norries SA (2020). Health system experiences of breast cancer survivors in urban South Africa. Womens Health (Lond).

[32] Ifeanyichukwu OA (2015). Assessment of breast cancer screening practices among women of Reproductive Age in Benin City, Edo state. International Journal of Tropical Disease and Health.

[33] Chatters LM (2000). Religion and Health: Public Health Research and Practice. Annu Rev Public Health.

